# Infrared Spectroscopic Imaging Visualizes a Prognostic Extracellular Matrix-Related Signature in Breast Cancer

**DOI:** 10.1038/s41598-020-62403-2

**Published:** 2020-03-25

**Authors:** Saumya Tiwari, Tiziana Triulzi, Sarah Holton, Viola Regondi, Biagio Paolini, Elda Tagliabue, Rohit Bhargava

**Affiliations:** 1 Departments of Medicine and Pharmacology, University of California San Diego, La Jolla, CA USA; 2 Molecular Targeting Unit, Department of Research, Fondazione IRCCS Istituto Nazionale dei Tumori, Milan, Italy; 3 University of Washington, Seattle, WA USA; 4 Anatomic Pathology A Unit, Department of Pathology, Fondazione IRCCS Istituto Nazionale dei Tumori, Milan, Italy; 5 Departments of Bioengineering, Electrical and Computer Engineering, Mechanical Science and Engineering, Chemical and Biomolecular Engineering and Chemistry, University of Illinois at Urbana-Champaign, Urbana, IL, USA; 6 Cancer Center at Illinois and Beckman Institute for Advanced Science and Technology, University of Illinois at Urbana–Champaign, Urbana, IL USA

**Keywords:** Analytical chemistry, Infrared spectroscopy

## Abstract

Molecular analysis techniques such as gene expression analysis and proteomics have contributed greatly to our understanding of cancer heterogeneity. In prior studies, gene expression analysis was shown to stratify patient outcome on the basis of tumor-microenvironment associated genes. A specific gene expression profile, referred to as ECM3 (Extracellular Matrix Cluster 3), indicated poorer survival in patients with grade III tumors. In this work, we aimed to visualize the downstream effects of this gene expression profile onto the tissue, thus providing a spatial context to altered gene expression profiles. Using infrared spectroscopic imaging, we identified spectral patterns specific to the ECM3 gene expression profile, achieving a high spectral classification performance of 0.87 as measured by the area under the curve of the receiver operating characteristic curve. On a patient level, we correctly identified 20 out of 22 ECM3 group patients and 19 out of 20 non-ECM3 group patients by using this spectroscopic imaging-based classifier. By comparing pixels that were identified as ECM3 or non-ECM3 with H&E and IHC images, we were also able to observe an association between tissue morphology and the gene expression clusters, showing the ability of our method to capture broad outcome associated features from infrared images.

## Introduction

Breast cancer is the most prevalent cancer in women^1^ and is estimated to cause over 40,000 deaths annually in the United States. Although cancer has been thought as a consequence of genetic mutations in an aberrant tissue mass, tumor progression is increasingly viewed as functionally interconnected with the surrounding microenvironment^2^. Indeed, crosstalk between cancer cells and the tumor microenvironment components [immune cells, stromal cells, and the extracellular matrix (ECM)] plays a major role in regulating several biological processes^3^. Differences in ECM-associated gene expression can describe the biological and clinical heterogeneity of breast cancer, especially in terms of prognosis and response to therapy^2^. Recent works have identified a subgroup of breast cancers characterized by overexpression of a robust cluster of genes mainly encoding structural ECM proteins, originally referred to as the ECM3 group^4,5^. These features indicate an epithelial to mesenchymal transition (EMT) but with accelerated metastatic potential only in the undifferentiated (grade III) phenotype^4,5^. Notably, ECM3 classification provides additional information to assess tumor progression^5^. It also identifies tumors in which the interplay between myeloid-derived suppressor cells (MDSCs) and the ECM drives the induction of EMT in tumor cells, and suggests a strategy for inhibition of MDSC by aminobisphosphonates that could revert EMT and lead to less aggressive tumor phenotype^6^.

Even though such molecular classification is of great importance in stratifying patients, it requires multi-step tissue processing and still requires the validation of an algorithm for classification at the single tumor level to be used instead of a clustering analysis on gene expression data of a cohort of samples. Additional information that could confirm this phenotype, without requiring multiple complicated steps or affecting the molecular integrity of the tissue, would be impactful. We thus sought a technology that could provide a molecular characterization of the stroma and provide this information in the context of the tumor to inform decision-making. Typical imaging techniques that could provide this context require labeling or other workflows that may not be compatible with established methods of genetic analyses of ECM3.

In this work, we evaluate an emerging technique for combined spatial and molecular analysis, infrared spectroscopic imaging, to map the downstream effects of this stromal gene expression onto the tissue. We then use this spectral information to determine outcome-associated patient groups. Fourier transform infrared (FT-IR) spectroscopic imaging is effective in providing spatially specific molecular analysis of samples for pathology, without the need for stains or dyes^7–11^. It can be used to probe biomolecular changes in the tumor microenvironment^8–10,12^ for comprehensive spatio-molecular assessment of the tissue which can be useful for determining prognosis^13^. We hypothesized that the differences in gene expression profiles between ECM3 and non-ECM3 tumors would also result in broad molecular composition changes in the tissue, which can be read with FT-IR spectroscopic imaging. Further, by associating gene expression profile with tissue molecular profiles, we can begin observing where on the tissue the downstream effects are the most prominent, thus spatially mapping the effect of altered gene expression.

## Results

### Classification of ECM3 and non-ECM3 tumors by FT-IR

**Figure 1:**
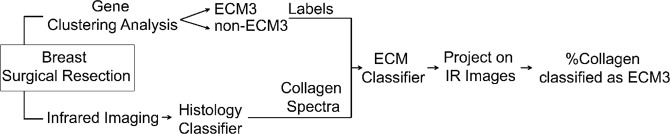
Summary of methods. In comparing IR imaging to gene expression profiling, we took a multi-step approach. Imaging was followed by an automated analysis of spectral data for each pixel using machine learning. From the assignment of cellular and acellular identity of all pixels in the image, collagen dominant pixels were analyzed. The spectral properties of these pixels were related to a label (ECM3, non-ECM3) for further analysis.

**Figure 2:**
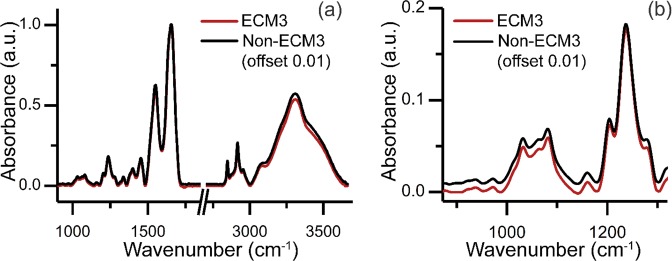
Spectral characteristics of collagen data in ECM3 and non-ECM3 tumors. (**a**) Mean absorbance across full spectral range. The average spectrum of the non-ECM3 group has been offset by adding 0.01 to the data for clarity. (**b**) Mean absorbance across spectral range between 872 cm^−1^ and 1320 cm^−1^ that was used for training and 5-fold cross validation. Non-ECM3 spectrum has been offset by adding 0.01 to the data for clarity.

Previous work showed high differences between ECM3 and non-ECM3 tumors at the transcriptional level, both according to ECM genes and to the whole transcriptional profile and based on the poor prognosis of patients with ECM3 grade III tumors^5^. In this work, we performed FT-IR spectroscopic imaging of 42 breast carcinomas, of which 22 were ECM3 and 20 were non-ECM3 as evaluated by gene expression profile analysis. Our goal was to find IR patterns that discriminate ECM3 from non-ECM3 tumors (Fig. 1), as molecular characteristics at the transcriptional level can be related to IR spectra^14,15^. Clinico-pathological characteristics of tumors analyzed are shown in Table 1. IR spectral analysis of tissues offers an exceptional opportunity to overcome tissue heterogeneity by generating a chemical map of the tissue. Instead of analyzing every pixel in the tissue without the context of tumor architecture, we first focused on determining the histologic identity of each pixel. This pixel-level identification is accomplished by applying machine learning to the acquired spectral data, as well described by numerous works for breast tissue^16–27^. Here, we first segmented the tissue image into histological components by applying a previously described histological classifier that labeled each pixel in the tissue section as one of the tissue classes i.e. epithelium, fibroblast, myofibroblast, collagen, blood, necrosis^28^. We found that when we used averaged IR spectra of each class to discriminate between ECM3 and non-ECM3 tumors, the highest discrimination performance was achieved with pixels classified in the collagen class (Supplementary Table 1). Studies have shown that the ECM in both patient^29^ and cultured samples^30^ is highly amenable to spectral analysis. Hence, we first examined average spectra, as shown in Fig. 2(a,b). Spectral differences between ECM3 and non-ECM3 in the collagen class are apparent in the region between 872 cm^−1^ and 1320 cm^−1^.We used these differences to develop the ECM3 classifier. We focused on this region also to limit the number of variables fed into the model and to prevent overfitting, especially given the small sample size. No significant differences in terms of signal to noise ratio (SNR) were observed among samples, even when collected over several months (Supplementary Fig. 1).

**Table 1:** Patient clinico-pathological characteristics according to ECM classification.

Characteristics	ECM3 (*N* = 22)	Non-ECM3 (*N* = 20)
**Median age**		
(years, range)	49 (36–78)	62 (47–82)
**Tumor size (cm)**		
<2 cm	14 (67%)	11 (55%)
≥2 cm	7 (33%)	9 (45%)
**Nodal Status**		
N0	4 (20%)	7 (35%)
N+	16 (80%)	13 (65%)
**ER status**		
Negative	2 (53%)	3 (57%)
Positive	20 (47%)	17 (43%)
**PGR status**		
Negative	2 (9%)	8 (40%)
Positive	20 (91%)	12 (60%)
**HER2 status**		
Negative	16 (73%)	17 (85%)
Positive	6 (27%)	3 (15%)
**Grade**		
I-II	12 (55%)	9 (45%)
III	10 (45%)	11 (55%)
**Chemotherapy treatment**		
No	2 (13%)	4 (20%)
Yes	13 (87%)	12 (80%)

### Development of IR imaging-based classifier to identify ECM3 tumors

**Figure 3:**
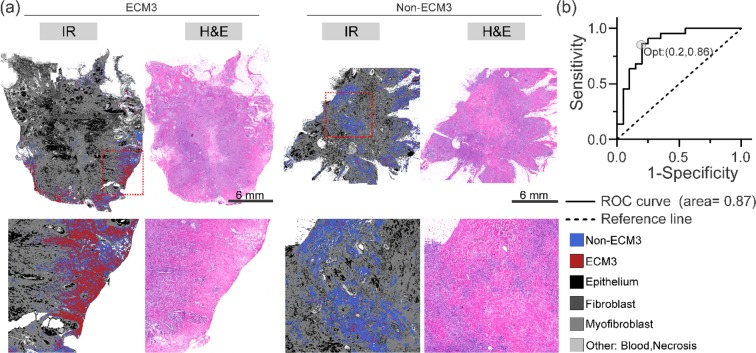
Results of 5-fold cross validated linear discriminant model: (**a**) Output of classifier as projected on complete surgical resection. Only collagen pixels were classified as either ECM3 or non-ECM3. Bottom row shows zoom-in sections from samples marked by red boxes. (**b**) Receiver operating characteristic curve for classifier output. ECM3 positive label was selected as positive class label. Posterior probability of classification was used for calculating false positive rate and true positive rate. Optimal operating point (Opt) was identified using the procedure described in methods.

The first 20 principal components were selected from the principal component analysis to develop the classifier. The principal components were calculated using only spectral band intensities from the collagen class within the range 872 cm^−1^–1320 cm^−1^. Utilizing these principal components, we trained a linear-discriminant classifier to categorize the average collagen spectrum from each tumor as either ECM3 or non-ECM3 (Fig. 3). The classifier performed well in discriminating ECM3 vs. non-ECM3 spectra, with an average area under the curve of the receiver operating characteristic (ROC) curve of 0.87 (95% CI: 0.76–0.98) for out-of-fold observations (Fig. 3b), using the optimal hyperparameter values (0.0001674 at 0.003 regularization parameter value). However, this result was only on the average spectra, demonstrating that the method provides accurate results, but also benefitting from the signal averaging process and likely susceptible to differences in sample sizes between samples. More importantly, it does not allow an evaluation of the spatial extent of the tumor. Hence, we sought next to evaluate the results of classification at the single-pixel level in each of the samples.

**Figure 4:**
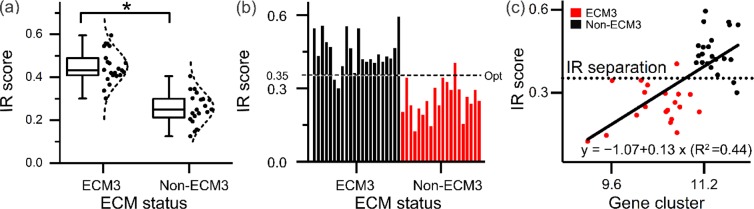
FT-IR based ECM3 discrimination model: (**a**) ECM3 positive pixel fraction (IR Score) in ECM3 and non-ECM3 patients as defined by gene expression analysis; *p-value less than 1.9 × 10^−10^. (**b**) IR based ECM3 classification can be performed by assigning ECM3 status to all tumors with IR score above Opt. (**c**) Linear model fit of expression of core genes of the ECM3 cluster with the IR score.

Every collagen pixel was classified as either ECM3 or non-ECM3 according to the spectral classifier (Fig. 3a) to evaluate tissue IR images. In Fig. 3a, several other histological components in the breast tissue are also identified in various shades of grey, which show correspondence to the serial sections’ H&E images. From the results of ECM-based classification, we observed that the majority of collagen pixels were classified as non-ECM3 in non-ECM3 tumors, while ECM3 tumors contained both non-ECM3 and a significant portion of ECM3 collagen pixels. This spatial heterogeneity arises from both a limitation of the predictive capability of numerical algorithms and may also arise from biological and intra-patient heterogeneity. While these images are useful in visualizing the extent of ECM3, a final decision regarding a patient testing positive requires abstraction of information to a binary decision at the patient level. To transfer pixels’ spectral level classification to the patient level, we defined the IR score as the ratio of collagen pixels identified as ECM3 to the total number of collagen pixels within the sample. This score was significantly higher in tumors identified as ECM3 as compared non-ECM3 tumors (Fig. 4a). To create a binary diagnostic test, we examined the distribution of the IR score for the patient population (Fig. 4b). At a separation threshold of about 0.35, the dichotomized IR score was able to accurately identify tumors belonging to ECM3 and non-ECM3 groups (Fig. 4b). At this cutoff, 20 out of 22 ECM3 tumors and 19 out of 20 non-ECM3 tumors were classified correctly (91% sensitivity, 95% specificity). Notably, a significant correlation between IR Score and the mean of log2 expression value of the 58 ECM3 genes, as obtained by gene expression data, was observed in the 42 tumor samples (Fig. 4c).

**Figure 5:**
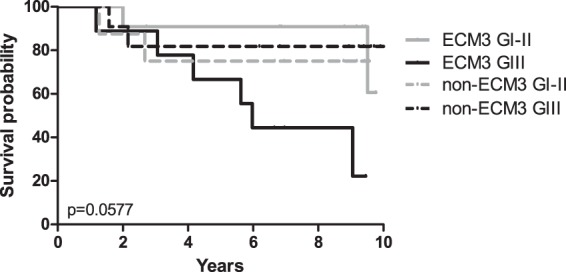
Association between ECM classification by IR score and grade with disease free survival. ECM3 grade III (ECM3 GIII, solid black line), non-ECM3 grade III (non-ECM3 GIII, dotted black line), ECM3 grade I-II (ECM3 GI-II, solid grey line) and non-ECM3 grade I-II (non-ECM3 GI-II, dotted grey line). p-value calculated by Log-rank test.

When we tested the linear fit of the continuous IR score with gene expression values, the model was able to explain about 45% variance in the gene expression results (R squared = 0.44). The slope of the linear fit model (0.13 ± 0.02) was significantly different from zero at p-value 8.9 e-7, indicating linear relationship between gene expression data and the IR imaging-based analysis (Table 2). In accordance with the high performance of FT-IR classifier, Kaplan Meier survival curves for the ECM groups determined by IR score showed a significant different survival in interaction with the tumor grade, as previously described^5^, confirming that patients with ECM3 grade III tumors had the worst prognosis (Fig. 5).

**Table 2:** Linear model fit of expression of core genes of the ECM3 cluster with the IR score.

		Value	Standard Error	t-Value	Prob >|t|
IR score	Intercept	−1.0658	0.24554	−4.34084	9.40E-05
IR score	Slope	0.13049	0.02248	5.80454	8.90E-07

### Identification of histological differences among ECM3 and non-ECM3 collagen pixels

**Figure 6:**
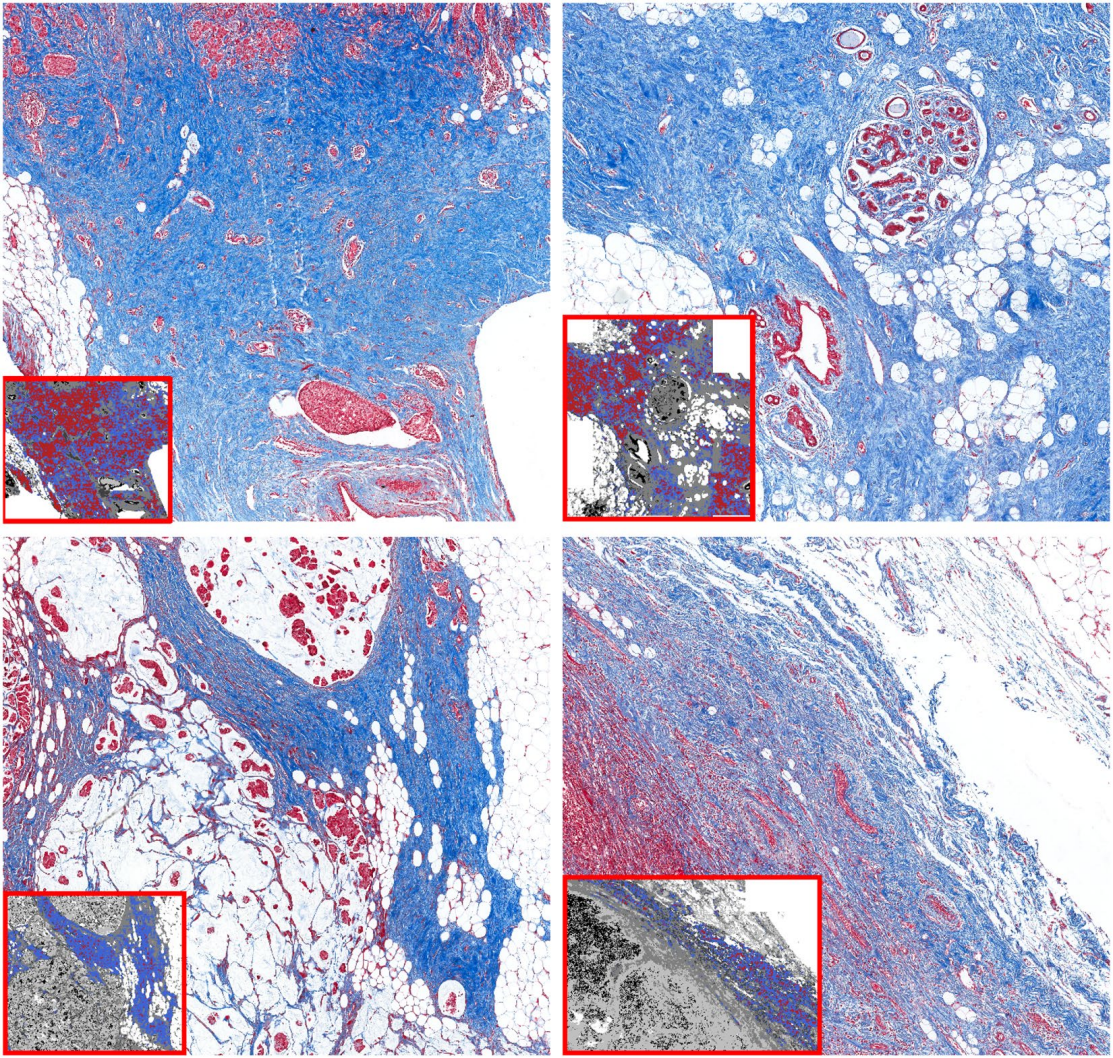
Collagen characteristics of ECM3 and non-ECM3 areas. Representative images of Masson’s trichrome staining in tumors classified as ECM3 (top panels) and non-ECM3 (bottom panels). In the small boxes, images of the collagen classifier at pixel level are shown. Red pixels: ECM3; blue pixels: non-ECM3.

While a correlative analysis of spectra with the ECM3 phenotype was observed, we were also interested in the origins of this correlation. Specifically, we investigated whether the specific chemical modification detected by IR spectral analysis in these areas could represent a difference in collagen fiber organization. Interestingly, we observed correspondence of the ECM3 and non-ECM3 regions with the density of collagen, which was also confirmed by Masson’s trichrome staining. ECM3 areas, which are mainly localized at the tumor borders, are characterized by dense wavy collagen bundles, whereas non-ECM3 pixels appear to correspond to areas with low collagen density and with sharp collagen fibers (Fig. 6). A relationship between spectral properties and physical organization is not surprising; however, we note that these types of powerful links between the traditional measures of pathology and emerging measures of molecular content are not common. The validation of staining patterns by spectral analysis shows that the identification of ECM3 can be made in terms of histologic modifications, yet without the use of stains, dyes or human interpretation.

In this work, we had a greater representation of T1 tumors with sizes less than 2 cm. Since tumors are quite heterogeneous, both ECM3 and non-ECM3 classified tumors showed ECM3-associated collagen, albeit in different proportions. We investigated whether the IR score reported here could be influenced by tumor size.

Although T1 tumors typically have higher median IR scores than T2 tumors (size greater than 2 cm), IR scores for both T1 and T2 appear to be within a similar range (Supplementary Fig. 2) indicating that our method identifies ECM3 tumors also in bigger, high heterogeneous tumors. This consistency is likely because our score considers only collagen areas and is normalized to total collagen content in the tissue, reducing variability due to heterogeneity. We are aware that we are not powered enough with our sample sizes to confirm this observation and further studies are warranted to validate it in bigger tumors and to determine from a biological point of view if ECM3 pixels are biologically relevant in inducing tumor aggressiveness Since ECM3 is prognostic in interaction with the grade, we also investigated IR scores according to tumor grade. IR scores appear to fall within similar ranges when compared between tumor grades (Supplementary Fig. 3(a)). Accordingly, using both grade and ECM categorization, we see significant differences between groups split by ECM classification, but not by tumor grade classification (Supplementary Fig. 3(b)). These results support the argument that IR scores based only on areas of collagen do not retain tumor grade effects.

## Discussion

In this work, we observed that the downstream effects of a specific gene expression signature can be read in from the tissue section using IR spectroscopic imaging. The ECM3 classification by gene expression used in this work is based on 738 genes that coded for the extracellular matrix proteins. ECM3 is characterized by overexpression of genes encoding mainly structural ECM protein (43 out of 58 genes characterizing ECM3 tumors), including several collagen chains (16 genes). Accordingly, a specific IR spectral pattern in the collagen area associated with the ECM3 gene expression profile. To our knowledge, this is the first indication that IR can visualize the product of altered gene expression in tissues. Considering that ECM3 and non-ECM3 tumors are classified based on the expression of ECM genes, an all-optical method to detect the same could be very useful. Given that there are differences in collagen spectra in the protein-rich region of the IR spectrum, it seems reasonable to expect that protein backbone signatures in IR could provide this discrimination. Indeed, most of the ECM3 genes (43/58) encode structural ECM proteins involved in the maintenance of connective tissue (collagens, laminins, and matrix-associated proteins), other than proteolytic enzymes. IR imaging is one of the few analytical methods able to provide information about collagen biodistribution and assembly^31^, without perturbing the tissue in any way and without the need for staining. We should also note that the stain-free ability of IR imaging to rapidly identify non-cellular and collagen-rich regions is a critical enabler of this recognition.

In this work, we identified a spectral predictor of ECM3 tumors in the collagen areas and the IR model explained 44% of the variability observed by gene expression. The gene expression analysis was carried out with frozen tissue samples, notably, while the IR imaging analysis was carried out with paraffin-embedded tissue sections. In addition, the areas used for both the techniques were adjacent but different, with the tissue needing to be crushed for gene expression analysis. Thus, we do expect some variability to arise from both processing as well as differences between the tissue sections. However, the concordance between the two is very exciting. Their observed relationship supports the ability of this technique in capturing the physiologic consequences of altered gene expression on the tissue, despite the substantial differences in the techniques that were used to probe the sample, as well as differences in sample preparation protocol.

We also utilized the spatio-biomolecular information obtained with IR spectroscopic imaging to visualize the biological effect derived from the altered gene expression in the tissue. By comparing the classified images to H&E and Masson’s trichrome images, we were able to see a correspondence between the changes in the collagen in the tumor microenvironment and the ECM classification by IR spectra. While it has recently been made possible to spatially map specific gene expression by amplifying the gene in small fragments of tissue^32^, in addition to conventional methods of measuring protein expression with IHC, there are no techniques to visualize the effects of a group of genes interacting together. From mapping the heterogeneity derived from the overexpression of the ECM-related genes, we observed that the stroma in which the ECM3 tumor is developing is different from non-ECM3 tumors at biochemical or structural organization levels.

Further analyses are needed to dissect the specific molecules or chemical/biological modification that explain the observed IR spectrum. However, it is conceivable that differences between ECM3 and non-ECM3 tumors are derived from the presence of different collagen types and/or in their organization in the microenvironment. Indeed, IR analysis has been previously described by several groups to differentiate the presence of different collagen types by their secondary structure parameters^31^. While most of IR absorptions are very similar between collagen types, a combination of signals from 4 spectral intervals (1700–1600; 1480–1350; 1300–1180; 1100–1005) was demonstrated to generate a robust classifier of collagen type^31^. These data suggest that a different ratio of collagen types in the area of collagen fibrils could be the basis of the ECM3 classifier. Collagen VI amount in the TME could likely be relevant for ECM3 classification by FT-IR. This indication is reasonable considering that type VI collagen presents the most evident spectral difference among collagen types^31^ and that ECM3 signature is characterized by overexpression of genes transcribing the three chain of collagen VI (*COL6A1*, *COL6A2* and *COL6A3*)^5^ In addition, ECM3 tumors express higher level of collagen VI by immunohistochemistry than non-ECM3 tumors^5^. It is also possible that ECM3 FT-IR signals identify areas with differences in collagen organization. IR spectra for collagens in the region we analyzed (872–1320) are mainly due to hydroxyproline, proline and phenylalanine residues other than structural α helix and amide III residues^33^. These residues are relevant for collagen elasticity and stiffness^34^, indicating possible roles in collagen organization. This association is also likely considering the differences in the collagen organization we observed in ECM3 pixels compared to non-ECM3. The different collagen organization is likely to derive from overexpression of different fibrillar collagen chains (like collagen I, III and V) together with collagen VI, a highly disulfide cross-linked microfibrillar collagen described to modify the organization of the fibrillar collagens intertwining with them^35^.

Despite the clear distinction between the ECM3 and non-ECM3 groups, the primary cause of the poor prognosis of patients with ECM3 grade III tumors remains unexplained. In this work, we did not find differences in collagen spectra based on the tumor grade. It is possible that collagen organization is not different in ECM3 grade III compared to ECM3 grade I-II tumors but that undifferentiated (grade III) tumor cells sense the TME, as the collagen VI, reported to favor tumor growth and progression^36^ differently. It is also possible that collagen organization derived from the interaction between undifferentiated tumor cells (grade III) and ECM3 TME is behind tumor progression, as observed in general in breast cancer^37^, and that this peculiar organization is not readable with IR imaging in the collagen areas. Further studies are needed to dissect the molecular features of the ECM3 TME that are responsible for patients’ poor prognosis.

In conclusion, we were able to identify an optical spectral ECM3 classifier as a relatively easy method to identify ECM3 tumors. As a proof of concept, the IR-spectroscopic imaging method utilized here is based on conventional Fourier Transform infrared spectroscopic imaging instruments, requiring 10–24 hours of acquisition for every surgical resection between 1–5 cm^2^. However, the speed of acquisition can be improved by manifolds with the recent advancements in high-definition^38^ and high-speed^29,39–41^ IR spectroscopic imaging coupled with machine learning to perform image analysis and prediction. In addition, classification rendered by IR spectroscopic imaging is less processing-intensive, providing prediction in a much simpler workflow than the conventional gene expression analysis protocols. Although confirmatory studies on independent case series are needed to evaluate the reproducibility of this classifier, the optimal cut-off and its prognostic performance in interaction with tumor grade, this methodology may have direct implications for future clinical assessment on breast cancer patients.

## Methods

### Patient cohort

The 42 tumors analyzed in this study were selected among a cohort of 97 consecutive primary breast cancer patients treated at Fondazione IRCCS Istituto Nazionale dei Tumori of Milan (INT, Italy) and characterized through whole gene expression profile analysis on HumanHT-12-v3 expression Bead Chip by Illumina^42^. Data of ECM classification, analyzed by using the Large Average Submatricies (LAS) biclustering algorithm described in Triulzi *et al*.^5^ were retrieved from the study. Expression data are available in the Gene Expression Omnibus data repository (GEO) with accession number GSE59595.

All procedures were per the Helsinki Declaration. Biospecimens used for research consisted of leftover material of samples collected during standard surgical and medical approaches at INT. Samples were donated by patients to the Fondazione IRCCS Istituto Nazionale dei Tumori BioBank for research purposes, and aliquots were allocated to this study after approval by the Institutional Review Board and a specific request to the Independent Ethical Committee of the institutes. The use of tissue for this study was approved by the University of Illinois Institutional Review Board via project 06684.

### Histochemistry

Formalin-fixed paraffin-embedded (FFPE) slides sequential to that used for FT-IR analysis were stained with H&E and with Masson’s Trichrome Stain Kit (Agilent) for direct visualization of collagen fibers. Stained slides were digitized by a slide scanner (ImageScope XT, Aperio).

### FT-IR image processing

5 µm thick FFPE breast cancer samples were sectioned onto IR-transparent Barium Fluoride salt plates. Sections were deparaffinated by washing in xylene for 24 hours, changing the solvent every three hours. FT-IR spectroscopic images of the tissue sections were collected using a Perkin Elmer Spotlight 400 system, equipped with 16 elements linear focal plane array HgCdTe detector. For each pixel, four scans were averaged at 4 cm^−1^ spectral resolution and 6.25 µm pixel size.

Image processing was performed in ENVI-IDL 4.8 with in-built and in-house written algorithms^10,43^. Images were stitched from the image tiles, baseline corrected, and normalized. The infrared images were converted to spectral metrics by calculating peak heights, areas and locations and taking ratios of these variables. Using these metrics, a previously developed breast supervised classifier was applied to the IR data to identify areas classified as collagen^28^.

### FT-IR data analysis

Post identification of collagen areas, all further data analysis was performed in Matlab R2016a. Baseline corrected and normalized infrared spectrum was extracted from areas identified as collagen in the histology classifier. To ensure that the data collected over several months did not have significant deviations in terms of signal to noise ratio (SNR), SNR was calculated as the ratio of peak intensities of 1656 cm^−1^ and 1826 cm^−1^ bands and compared by student’s t-test. While it appears that there is no significant difference between the SNR of the groups, due to low power we added a spectral smoothening step by application of Savitzky-Golay 9-point filter to further ensure that SNR did not affect our analysis. Spectral data points between 872 cm^−1^ and 1320 cm^−1^ were used for training. Data were cleaned by removing out of range observations with absorbance values greater than 2 or less than −1. For the training set, the mean collagen spectra per patient were calculated. Principal components analysis was performed to reduce the data dimensionality, and the first 20 components were chosen for further study. To perform principal components analysis, we used the raw intensity values at each spectral band collected between 872 cm^−1^ and 1320 cm^−1^. Linear discriminant classification with 5-fold cross-validation was used to develop the ECM3 classifier to classify ECM3 and non-ECM3 spectra. Hyperparameters that minimized the 5-fold cross-validation loss were automatically determined by using Matlab’s automatic hyperparameter optimization protocols. From this, the optimal linear coefficient threshold Delta was determined to be 0.0001674 at 0.003 regularization parameter value. Delta threshold was used to determine the eligibility of variables for inclusion in the model, while the regularization parameter was used to prevent overfitting. Post ECM3 classification, the classifier was projected onto the full IR images and the total number of pixels classified as ECM3 and non-ECM3 were calculated. The percentage of ECM3 pixels out of the total number of collagen pixels was used to stratify patient groups. These values were fit in a logistic regression model to identify the optimal cutoff point for patient stratification.

### Statistical analysis

Fisher’s exact test tested association among categorical variables. Two-sided p < 0.05 was considered significant. Survival functions were assessed using the Kaplan-Meier estimator, while the log-rank test was used to compare survival distributions.

## Supplementary information

Supplementary information.

## Data Availability

Gene expression data used in this study are available in the Gene Expression Omnibus data repository (GEO) with accession number GSE59595. The IR datasets generated during the current study are available from the corresponding author upon request.

## References

[1] CDC - Breast Cancer Statistics. Available at, https://www.cdc.gov/cancer/breast/statistics/index.htm (Accessed: 13th August 2018).

[2] Giussani M, Merlino G, Cappelletti V, Tagliabue E, Daidone MG (2015). Tumor-extracellular matrix interactions: Identification of tools associated with breast cancer progression. Semin. Cancer Biol..

[3] Quail DF, Joyce JA (2013). Microenvironmental regulation of tumor progression and metastasis. Nat. Med..

[4] Bergamaschi A (2008). Extracellular matrix signature identifies breast cancer subgroups with different clinical outcome. J. Pathol..

[5] Triulzi T (2013). Neoplastic and stromal cells contribute to an extracellular matrix gene expression profile defining a breast cancer subtype likely to progress. PLoS One.

[6] Sangaletti S (2016). Mesenchymal Transition of High-Grade Breast Carcinomas Depends on Extracellular Matrix Control of Myeloid Suppressor Cell Activity. Cell Rep..

[7] Fernandez DC, Bhargava R, Hewitt SM, Levin IW (2005). Infrared spectroscopic imaging for histopathologic recognition. Nat. Biotechnol..

[8] Holton SE, Bergamaschi A, Katzenellenbogen BS, Bhargava R (2014). Integration of molecular profiling and chemical imaging to elucidate fibroblast-microenvironment impact on cancer cell phenotype and endocrine resistance in breast cancer. PLoS One.

[9] Mayerich, D. *et al*. Stain-less staining for computed histopathology. *Technology* 1–5, 10.1142/S2339547815200010 (2015). 10.1142/S2339547815200010 PMC4445956 26029735

[10] Baker MJ (2014). Using Fourier transform IR spectroscopy to analyze biological materials. Nat. Protoc..

[11] Bhargava R, Fernandez DC, Hewitt SM, Levin IW (2006). High throughput assessment of cells and tissues: Bayesian classification of spectral metrics from infrared vibrational spectroscopic imaging data. Biochim. Biophys. Acta.

[12] Holton SE, Walsh MJ, Bhargava R (2011). Subcellular localization of early biochemical transformations in cancer-activated fibroblasts using infrared spectroscopic imaging. Analyst.

[13] Kwak JT (2015). Improving Prediction of Prostate Cancer Recurrence using Chemical Imaging. Sci. Rep..

[14] Smolina M, Goormaghtigh E (2018). Gene expression data and FTIR spectra provide a similar phenotypic description of breast cancer cell lines in 2D and 3D cultures. Analyst.

[15] Smolina M, Goormaghtigh E (2015). Infrared imaging of MDA-MB-231 breast cancer cell line phenotypes in 2D and 3D cultures. Analyst.

[16] Fabian H, Lasch P, Boese M, Haensch W (2002). Mid-IR microspectroscopic imaging of breast tumor tissue sections. Biopolymers.

[17] Zhang L, Small GW, Haka AS, Kidder LH, Lewis EN (2003). Classification of Fourier Transform Infrared Microscopic Imaging Data of Human Breast Cells by Cluster Analysis and Artificial Neural Networks. Appl. Spectrosc..

[18] Mohamed HT (2018). Characterization of inflammatory breast cancer: a vibrational microspectroscopy and imaging approach at the cellular and tissue level. Analyst.

[19] Ali MHM, Rakib F, Al-Saad K, Al-Saady R, Goormaghtigh E (2019). An Innovative Platform Merging Elemental Analysis and Ftir Imaging for Breast Tissue Analysis. Sci. Rep..

[20] Fabian H (2006). Diagnosing benign and malignant lesions in breast tissue sections by using IR-microspectroscopy. Biochim. Biophys. Acta.

[21] Bénard, A. *et al*. Discrimination between healthy and tumor tissues on formalin-fixed paraffin-embedded breast cancer samples using IR imaging. *J. Spectrosc*. **24**, 67–72 (2010).

[22] Walsh MJ, Kajdacsy-Balla A, Holton SE, Bhargava R (2012). Attenuated total reflectance Fourier-transform infrared spectroscopic imaging for breast histopathology. Vib. Spectrosc..

[23] Clède S, Policar C, Sandt C (2014). Fourier Transform Infrared (FT-IR) Spectromicroscopy to Identify Cell Organelles: Correlation with Fluorescence Staining in MCF-7 Breast Cancer Cells. Appl. Spectrosc..

[24] Depciuch J (2016). Application of Raman Spectroscopy and Infrared Spectroscopy in the Identification of Breast Cancer. Appl. Spectrosc..

[25] Chrabaszcz K (2018). FT-IR- and Raman-based biochemical profiling of the early stage of pulmonary metastasis of breast cancer in mice. Analyst.

[26] Ali MH (2018). A simple model for cell type recognition using 2D-correlation analysis of FTIR images from breast cancer tissue. J. Mol. Struct..

[27] Kar S, Katti DR, Katti KS (2019). Fourier transform infrared spectroscopy based spectral biomarkers of metastasized breast cancer progression. Spectrochim. Acta Part A Mol. Biomol. Spectrosc..

[28] Mayerich, D. M., Walsh, M., Kadjacsy-Balla, A., Mittal, S. & Bhargava, R. Breast histopathology using random decision forests-based classification of infrared spectroscopic imaging data. In (eds. Gurcan, M. N. & Madabhushi, A.) 904107, 10.1117/12.2043783 (SPIE, 2014).

[29] Mittal S (2018). Simultaneous cancer and tumor microenvironment subtyping using confocal infrared microscopy for all-digital molecular histopathology. Proc. Natl. Acad. Sci. USA.

[30] Smolina M, Goormaghtigh E (2016). FTIR imaging of the 3D extracellular matrix used to grow colonies of breast cancer cell lines. Analyst.

[31] Belbachir K, Noreen R, Gouspillou G, Petibois C (2009). Collagen types analysis and differentiation by FTIR spectroscopy. Anal. Bioanal. Chem..

[32] Ganguli A (2018). Pixelated spatial gene expression analysis from tissue. Nat. Commun..

[33] Gąsior-Głogowska M, Komorowska M, Hanuza J, Ptak M, Kobielarz M (2010). Structural alteration of collagen fibres–spectroscopic and mechanical studies. Acta Bioeng. Biomech..

[34] Ghanaeian A, Soheilifard R (2018). Mechanical elasticity of proline-rich and hydroxyproline-rich collagen-like triple-helices studied using steered molecular dynamics. J. Mech. Behav. Biomed. Mater..

[35] Gelse K, Pöschl E, Aigner T (2003). Collagens—structure, function, and biosynthesis. Adv. Drug Deliv. Rev..

[36] Chen P, Cescon M, Bonaldo P (2013). Collagen VI in cancer and its biological mechanisms. Trends Mol. Med..

[37] Conklin MW, Keely PJ (2012). Why the stroma matters in breast cancer. Cell Adh. Migr..

[38] Reddy RK, Walsh MJ, Schulmerich MV, Carney PS, Bhargava R (2013). High-definition infrared spectroscopic imaging. Appl. Spectrosc..

[39] Yeh, K. & Bhargava, R. Discrete frequency infrared imaging using quantum cascade lasers for biological tissue analysis. In (eds. Mahadevan-Jansen, A. & Petrich, W.) 9704, 970406 (International Society for Optics and Photonics, 2016).

[40] Yeh K, Kenkel S, Liu J-N, Bhargava R (2015). Fast Infrared Chemical Imaging with a Quantum Cascade Laser. Anal. Chem..

[41] Tiwari, S., Raman, J., Reddy, V., Dawson, M. & Bhargava, R. Translation of infrared chemical imaging for cardiovascular evaluation. In SPIE BiOS (eds. Mahadevan-Jansen, A. & Petrich, W.) 97040X, 10.1117/12.2230004 (International Society for Optics and Photonics, 2016).

[42] Huang X (2015). Molecular portrait of breast cancer in China reveals comprehensive transcriptomic likeness to Caucasian breast cancer and low prevalence of luminal A subtype. Cancer Med..

[43] Tiwari S, Bhargava R (2015). Extracting knowledge from chemical imaging data using computational algorithms for digital cancer diagnosis. Yale J. Biol. Med..

