# Supplementary material for: Infrared Spectroscopic Imaging Visualizes a Prognostic Extracellular Matrix-Related Signature in Breast Cancer
Source: Sci Rep. 2020 Mar 25;10:5442. doi: 10.1038/s41598-020-62403-2 (PMC7096505; doi:10.1038/s41598-020-62403-2)
Supplement: Supplementary file 1 — Supplementary information. [file 41598_2020_62403_MOESM1_ESM.docx]

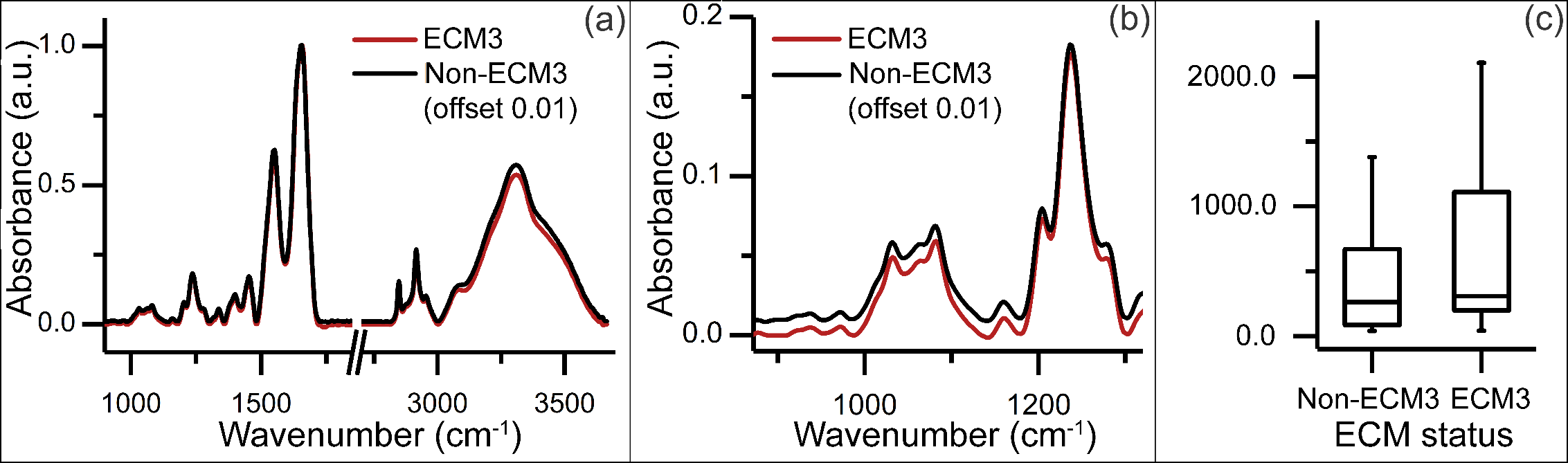


Supplementary figure 1: Comparison of signal to noise ratio, calculated as ratio of absorbance at 1656 cm^-1^ to 1826 cm^-1^. From two-sample t-test, no significant difference was noted in the SNR between the two groups at 0.14 power.


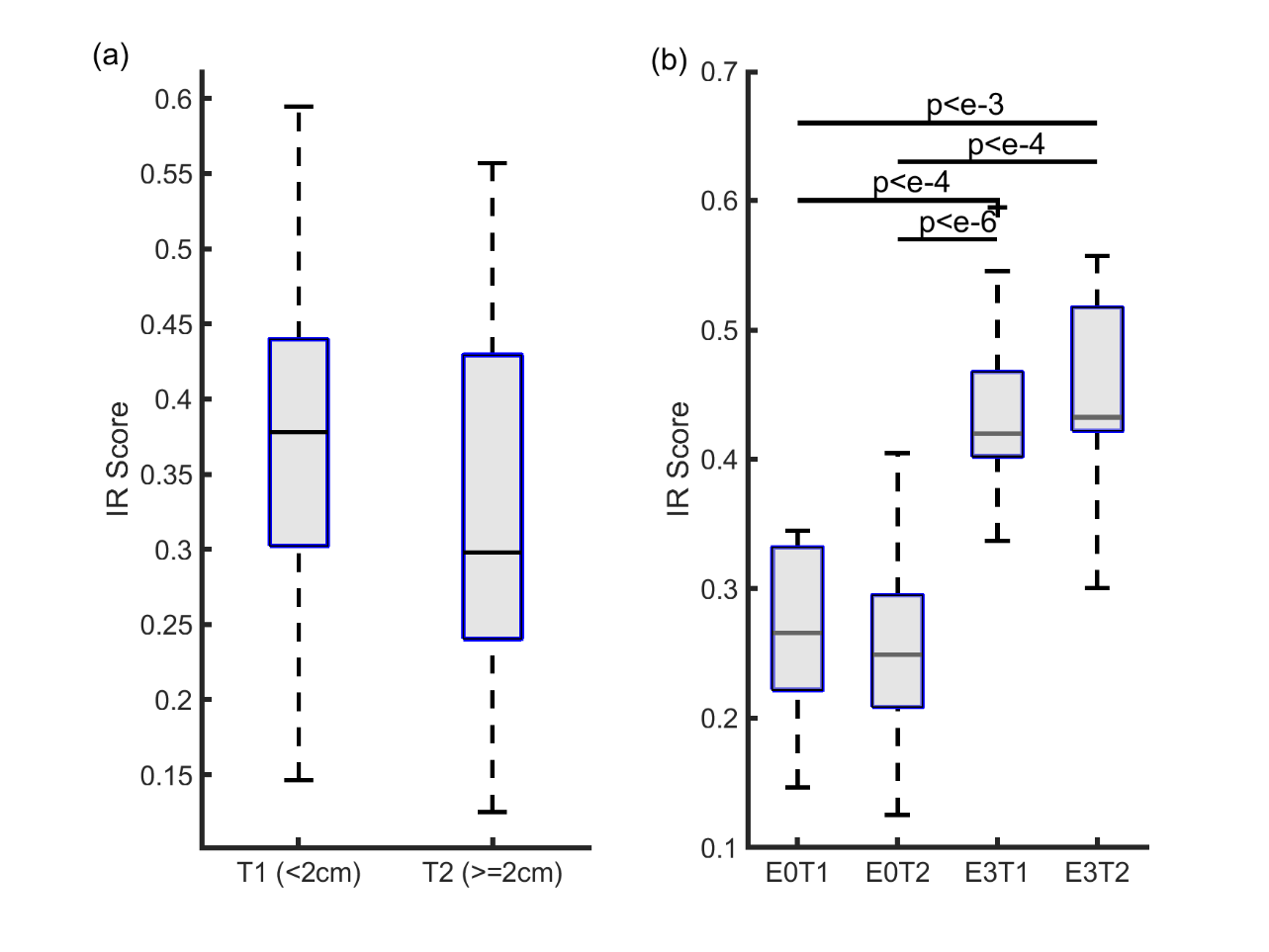


Supplementary Figure 2: Variation of IR Scores with tumor sizes. (a) IR score distribution between T1 and T2 tumors. (b) IR score distribution between T1 and T2 tumors split by ECM3 tumors (E3) and non ECM3 tumors (E0)


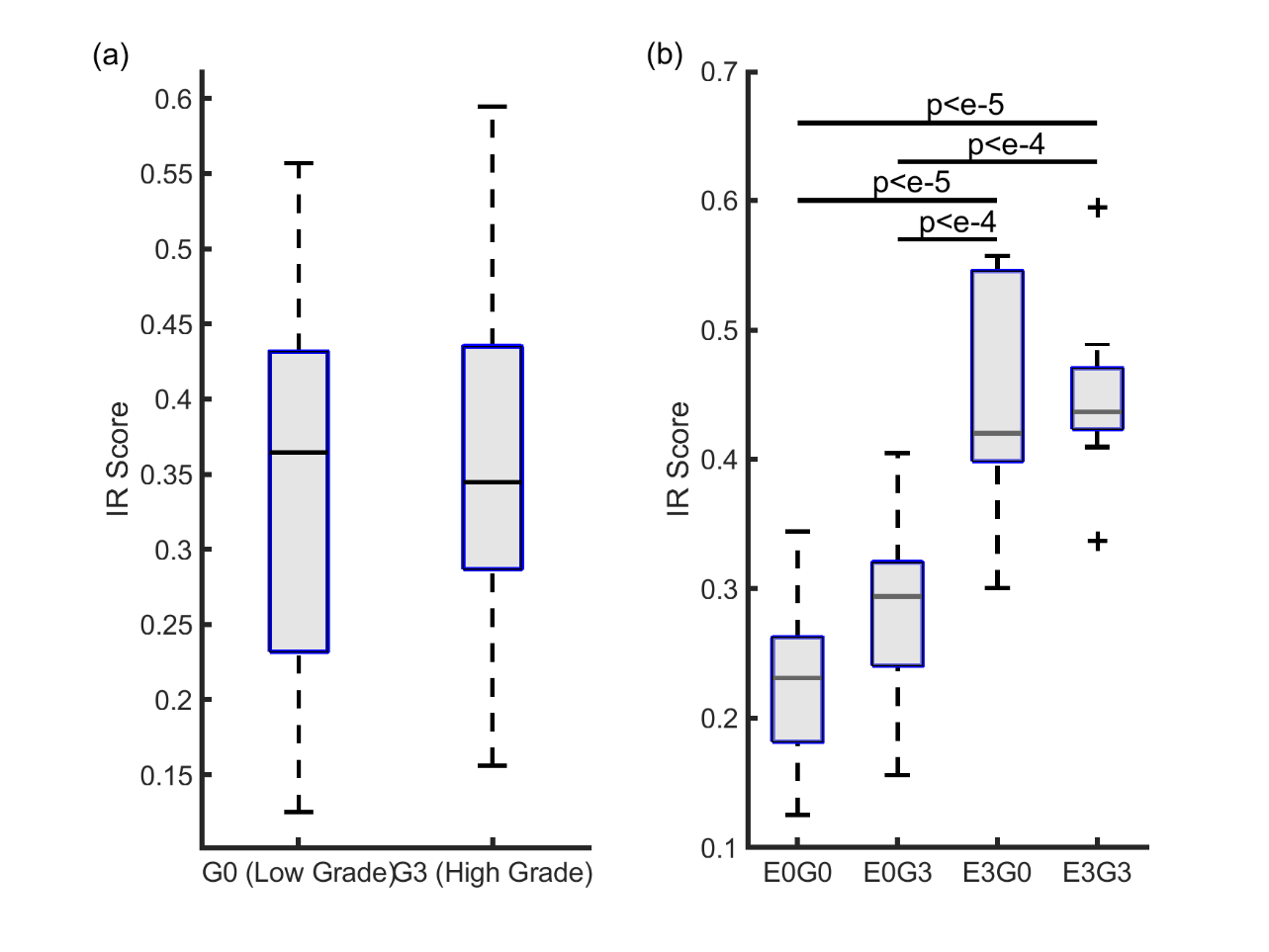


Supplementary figure 3: Variation of IR Scores with tumor grade. (a) IR score distribution between G0 (low grade) and G3 (high grade) tumors. (b) IR score distribution between G0 and G3 tumors split by ECM3 tumors (E3) and non ECM3 tumors (E0)

**Supplementary Table 1. Predictive performance of the histological classifiers in identifying ECM3 tumors**

| **Class** | **Training**  **AUC** | **Calibration AUC** | **Validation AUC** |
| --- | --- | --- | --- |
| Epithelium | 0.85 | 0.86 | 0.62 |
| Fibroblast | 0.94 | 0.87 | 0.77 |
| Myofibroblast | 0.70 | 0.67 | 0.72 |
| Collagen | 0.97 | 0.95 | 0.87 |
| Blood | 0.81 | 0.82 | 0.63 |

AUC: Area under the ROC curve
